# Predicting acute kidney injury in severe trauma. A biomarker breakthrough?

**DOI:** 10.1186/s13054-015-1150-z

**Published:** 2015-12-26

**Authors:** Patrick M. Honore, Rita Jacobs, Inne Hendrickx, Elisabeth De Waele, Viola Van Gorp, Herbert D. Spapen

**Affiliations:** Intensive Care Unit Department, Universitair Ziekenhuis Brussel, Vrije Universiteit Brussel, 101, Laarbeeklaan, Jette, Brussels 1090 Belgium

We read with great interest the recent article by Stewart et al. highlighting that urinary biomarkers of acute kidney injury (AKI) could identify combat injury patients at high risk of dying or requiring renal replacement therapy (RRT) [1].

According to recent in-depth reviews, the application of biomarkers to predict AKI in critically ill patients is cumbersome at best [2, 3]. Biomarkers have poor discriminative power in heterogeneous patient populations, and levels are significantly influenced by systemic inflammation, pre-existing renal failure, and timing of sampling. Within this context, the study by Stewart et al. is both refreshing and challenging. It focuses on severely injured patients, a fairly well-defined population at substantial risk for developing early AKI [4, 5], by using a biomarker panel that consists mainly of upregulated proteins that are most sensitive to detect true histological kidney damage. Whereas the positive predictive value of these biomarkers would argue against urgent evacuation of combat casualties, intensive care unit physicians evidently will adhere an opposite “triage” and invest in a more early and intensive approach of imminent AKI.

The data provided by Stewart et al. also allow a step forward in creating a more performant biomarker-inclusive model (or score?) for predicting the development of AKI in critically ill trauma patients.

A logical next step would be to evaluate the impact of different RRT techniques (e.g., intermittent versus continuous RRT) on morbidity and mortality in this population.

## Abbreviations

AKI: acute kidney injury
RRT: renal replacement therapy
