# Supplementary material for: Predicting acute kidney injury in severe trauma. A biomarker breakthrough?
Source: Crit Care. 2015 Dec 26;19:432. doi: 10.1186/s13054-015-1150-z (PMC4699345; doi:10.1186/s13054-015-1150-z)
Supplement: Related texts

# Reply: Authors’ response

**Authors:** Jonathan A. Sosnov, Ian J. Stewart, Kevin K. Chung

We agree with the analysis by Honore et al. on the future of biomarker research. The current method for diagnosing AKI, via changes in creatinine, is clearly inadequate. One reason for this is the significant lag function from the insulting event. This and the other well-known limitations of creatinine as a biomarker resulted in the search for urinary biomarkers. Although our study did not have sufficient power to create a biomarker-inclusive model to clinically predict AKI and its outcomes, our work adds to the literature that argues for its development [1]. Such a model of AKI would give us a context to develop effective treatments and better allow us to power randomized trials. We do not know how to optimally modulate fluid replacement [6]. We do not know the optimal type or timing of RRT [7]. If we could use an inclusive model to diagnose AKI earlier and correlate changes in urinary biomarkers to specific subsequent outcomes, we might be able to design better intervention studies to answer these important questions.
